# Machine learning models for predicting one-year survival in patients with metastatic gastric cancer who experienced upfront radical gastrectomy

**DOI:** 10.3389/fmolb.2022.937242

**Published:** 2022-12-01

**Authors:** Cheng Zhang, Yi Zhang, Ya-Hui Yang, Hui Xu, Xiao-Peng Zhang, Zhi-Jun Wu, Min-Min Xie, Ying Feng, Chong Feng, Tai Ma

**Affiliations:** ^1^ Department of Oncology, The First Affiliated Hospital of Anhui Medical University, Hefei, Anhui, China; ^2^ Anhui Provincial Cancer Institute/Anhui Provincial Office for Cancer Prevention and Control, Hefei, Anhui, China; ^3^ Department of Noncommunicable Diseases and Health Education, Hefei Center for Disease Control and Prevention, Hefei, Anhui, China; ^4^ Department of Oncology, Ma’anshan Municipal People’s Hospital, Ma’anshan, Anhui, China

**Keywords:** stomach neoplasms, neoplasm metastasis, survival analysis, supervised machine learning, electronic medical record, clinical laboratory information system

## Abstract

Tumor metastasis is a common event in patients with gastric cancer (GC) who previously underwent curative gastrectomy. It is meaningful to employ high-volume clinical data for predicting the survival of metastatic GC patients. We aim to establish an improved machine learning (ML) classifier for predicting if a patient with metastatic GC would die within 12 months. Eligible patients were enrolled from a Chinese GC cohort, and the complete detailed information from medical records was extracted to generate a high-dimensional dataset. Appropriate feature engineering and feature filter were conducted before modeling with eight algorithms. A 10-fold cross validation (CV) nested in a holdout CV (8:2) was employed for hyperparameter tuning and model evaluation. Model selection was based on the area under the receiver operating characteristic (AUROC) curve, recall, and precision. The selected model was globally explained using interpretable surrogate models. Of the total 399 cases (median survival of 8.2 months), 242 patients survived less than 12 months. The linear discriminant analysis (LDA), support vector machine (SVM), and random forest (RF) model had the highest AUROC (0.78 ± 0.021), recall (0.93 ± 0.031), and precision (0.80 ± 0.026), respectively. The LDA model created a new function that generally separated the two classes. The predicted probability of the SVM model was interpreted using a linear regression model visualized by a nomogram. The predicted class of the RF model was explained using a decision tree model. In summary, analyzing high-volume medical data by ML is helpful to produce an improved model for predicting the survival in patients with metastatic GC. The algorithm should be carefully selected in different practical scenarios.

## Introduction

Gastrectomy with adequate lymphadenectomy provides a potential opportunity of cure for resectable gastric cancer (GC) (Smyth et al., 2020); however, a substantial proportion of patients still develop recurrence or metastasis afterward (Chen et al., 2021; Hisamori et al., 2021). The prognosis of metastatic GC is expected to be poor; the survival time after relapse varies from 3–15 months (Smyth et al., 2020), depending on the metastatic site (Chau et al., 2004; Lee et al., 2007; Kim et al., 2008; Custodio et al., 2017), performance status (Chau et al., 2004; Lee et al., 2007; Kim et al., 2008; Custodio et al., 2017), palliative chemotherapeutic regimen, and other factors (Custodio et al., 2017; Zhu et al., 2022). Several models have been established based on clinical trials or real-world data, aiming to precisely estimate the survival probability in these patients (Chau et al., 2004; Lee et al., 2007; Kim et al., 2008; Koo et al., 2011; Custodio et al., 2017). Although different sets of variables have been incorporated, the ability of survival prediction in the traditional model is dissatisfactory. A Spanish multicenter study (the AGAMENON study) developed a nomogram-based model to predict the survival of patients with advanced GC (AGC), with an accuracy of 0.67 in the validation set (Custodio et al., 2017). A Korean single-center study constructed a score-based model with an accuracy of 0.58 (Koo et al., 2011), then externally validated another three models (Chau et al., 2004; Lee et al., 2007; Kim et al., 2008), and showed similar performances in the same population (Koo et al., 2011). Our previous work also developed a score-based model in a Chinese cohort with a c-index of 0.67 (Ma et al., 2021). Meanwhile, we validated seven published models (Chau et al., 2004; Lee et al., 2007; Kim et al., 2008; Koo et al., 2011; Takahari et al., 2014; Wang et al., 2016; Kim et al., 2020) in a Chinese population, and the results showed that the area under receiver operating characteristic (AUROC) curves was only about 0.60 (Xu et al., 2021).

The traditional prognostic model is frequently built by the logistic or Cox regression analysis on the basis of the well-known clinical and pathological variables, for example, performance status (Chau et al., 2004; Lee et al., 2007; Kim et al., 2008; Takahari et al., 2014), tumor differentiation (Custodio et al., 2017; Kim et al., 2020), metastatic sites (Kim et al., 2008; Takahari et al., 2014; Wang et al., 2016), and routine laboratory tests (Koo et al., 2011; Custodio et al., 2017; Kim et al., 2020; Ma et al., 2021). The selection of candidate variables is typically guided by the clinical experience and previous literature. In the era of digital medicine, the electronic medical record (EMR) and laboratory information system (LIS) make massive medical data readily available; nevertheless, we are still far from taking full advantage of them. One possible reason is the incompetence of the classic statistical method in dealing with numerous independent variables, which emphasizes the need for adopting a new strategy of statistics.

Machine learning (ML) is increasingly used for data mining due to its capacity to tackle big data. In order to utilize the abundant digital medical records and further improve model performance in predicting the survival of GC patients with recurrence or metastasis after radical gastrectomy, we enroll eligible participants from a retrospective GC cohort, build a high-dimensional dataset from the EMR and LIS, identify the most relevant prognostic factors, and implement modeling using several ML algorithms.

## Materials and methods

### Study setting and population

**FIGURE 1:**
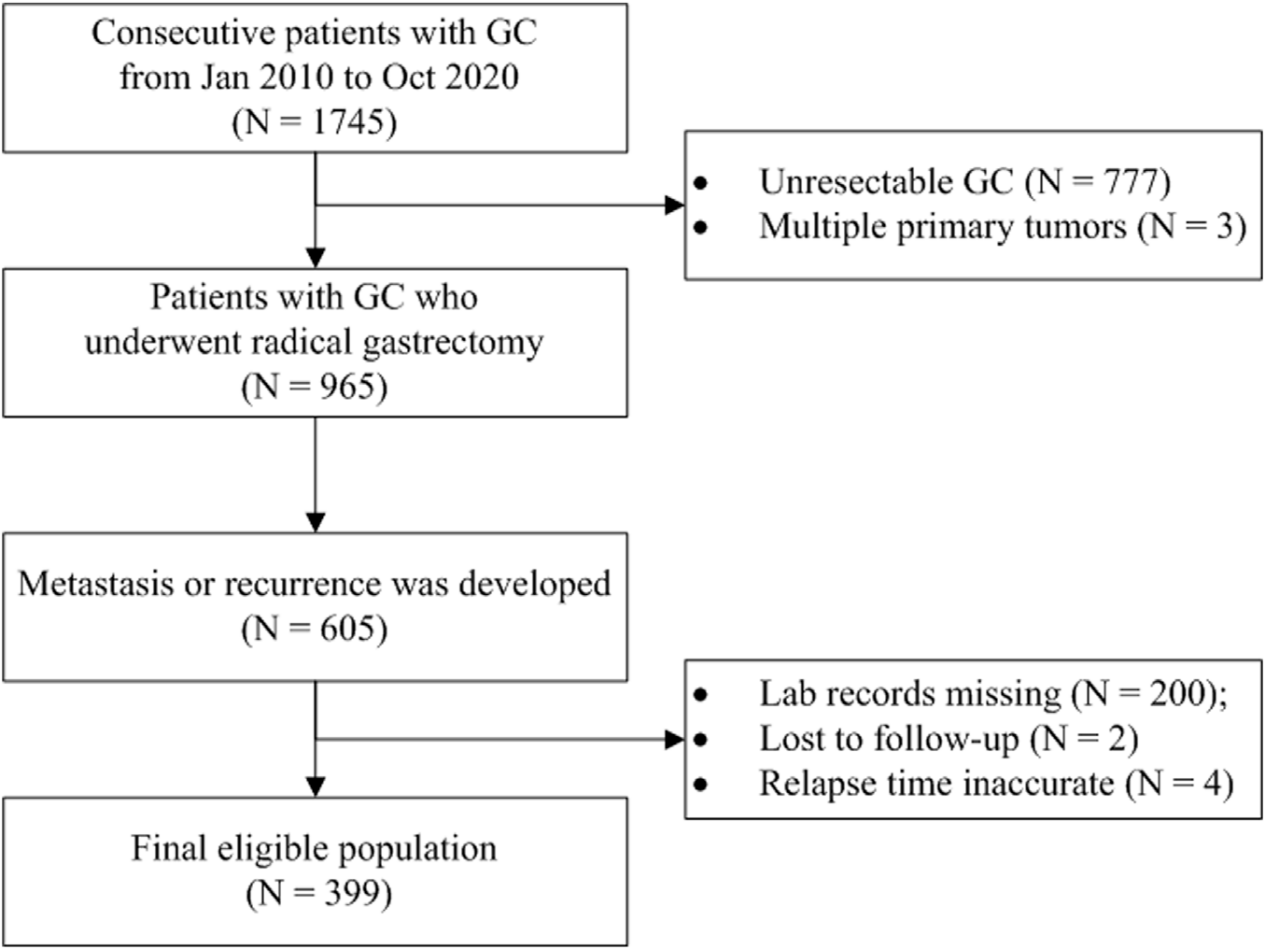
Flow diagram of patient selection. Abbreviation: GC, gastric cancer.

In this retrospective study, we trained ML models using different algorithms to predict if a GC patient would die within 12 months after the first metastasis or recurrence because 12 months is typically recognized as the median survival time for patients with AGC (Smyth et al., 2020). The participants were enrolled from a registered hospital-based GC cohort (ChiCTR1800019978, http://www.chictr.org.cn/). The consecutive gastric or esophagogastric junction carcinoma patients who underwent radical gastrectomy and developed disease recurrence or metastasis were included and followed up in the cohort. Those patients with multiple primary malignant tumors or with no records of laboratory examinations at the time of metastasis were excluded. The EMR and LIS were retrieved to obtain data for analysis. The survival information was acquired from the death register system or by telephonic follow-up conducted every 3 months. The overall survival (OS) was defined as the interval between the first metastasis and death or the last follow-up. The workflow of the study is illustrated in Figure 1.

All procedures performed in the study involving human participants were in accordance with the 1964 Helsinki Declaration and its later amendments or comparable ethical standards. The studies involving human participants were reviewed and approved by the Ethics Committee of The First Affiliated Hospital of Anhui Medical University (reference number: Quick-PJ-2021-05-19). The Ethics Committee waived the requirement of written informed consent for participation.

### Dataset and feature engineering

All features are listed in Supplementary Table S1. Briefly, the dataset included information about demography, histopathology, surgical resection, postoperative adjuvant chemotherapy, first-line palliative chemotherapy, radiotherapy, baseline laboratory records at the time of metastasis (routine blood test, biochemistry, coagulation, immunology, and tumor biomarkers), and survival. Each aspect had several items to record the details, so a high-dimensional dataset was generated.

Categorical features were transformed by one-hot encoding. Numerical features were standardized, normally transformed, or grouped where appropriate. In our dataset, missing values generally occurred at random, so they were deleted (the fraction of the missing values over the total cases was more than 30%) or imputed using decision tree algorithm.

ML model performance may suffer from high dimensionality, so, here, some features were filtered out prior to modeling. A feature with zero or near-zero variance was first dropped because it provided no useful information to a model. The rule of detecting a near-zero variance feature was (Smyth et al., 2020) that the fraction of unique values over the sample size was less than 10% and (Hisamori et al., 2021) the ratio of the frequency of the most prevalent value to the frequency of the second most prevalent value was more than 20% (Boehmke and Greenwell, 2019). Next, we used the importance value calculated by the random forest (RF) algorithm to rank the features and select a number of them that contributed most to the model. The specific number was tuned by a random search during model development. All these data-dependent preprocessings were conducted in isolation of each resampling iteration in order to avoid data leakage.

### Model development

First, the entire dataset was randomly split into a training set and a validation set (8:2) as the outer layer. Then, the training set was further randomly split by 10-fold cross-validation (CV) as the inner layer. The inner layer was used to tune hyperparameters by random search, and the best configuration was passed on the validation set from the outer layer to evaluate the model performance. The nested CV design reduced the risk of overestimation of the model because the information of the training set was not leaked into the validation set. The whole process was repeated five times for averaging the effect of randomness, so we used the mean value to measure the model performance. The AUROC curve was the primary indicator to evaluate the model because it did not have any bias toward classifiers on balanced or imbalanced binary prediction problems (He and Ma, 2013). Precision and recall were also crucial as they reflected the false-positive error and the false-negative error of the model, respectively. In addition, accuracy and F1 score (the harmonic mean of precision and recall) were also calculated.

We used eight common classification algorithms for modeling: kernel K-nearest neighbor (KKNN), linear discriminant analysis (LDA), support vector machine (SVM), RF, XGBoost, ridge regression, LASSO regression, and elastic net regression. For each algorithm, the hyperparameters that needed to be tuned and the optimal settings are given in Supplementary Table S2. The whole project was deployed using RStudio 1.4.1717 with packages “mlr3verse” (modeling pipeline and framework), “kknn” (KKNN algorithm), “e1071” (SVM algorithm), “MASS” (LDA algorithm), “ranger” (RF algorithm), “xgboost” (XGBoost algorithm), and “glmnet” (ridge, LASSO, and elastic net regression). To make a comparison with the traditional method, we used the logistic regression as a reference algorithm.

A learning curve is used to diagnose if the sample size is adequate for modeling and if an overfitting or underfitting problem occurs. It comprises two lines that represent the errors of the training set and the validation set, respectively, in relation to the sample size. The training learning curve shows how well the model is learning, and the validation curve shows how well the model is generalizing. If a model is underfitting, the error of the training set is close to that of the validation set, so obtaining more samples is unlikely to improve the performance. In contrast, if a model is overfitting, the gap between the errors of the training set and validation set is large, so adding more samples is likely to be helpful.

### Model interpretation

Only the selected models were interpreted, which comprised the LDA, the SVM, and the RF-based model. The model interpretation was based on the final model built on the entire dataset with the tuned hyperparameters or model configuration. The general theories of the three algorithms were briefly demonstrated. The LDA aims to learn a new line, called the discriminant function (DF) that combines the original features in a linear fashion, weighting greater for “better” predictors and less for “poorer” predictors. The value that gives the weight for each feature is called the DF coefficient, which indicates how much it contributes to class discrimination. The DF separates the centroid of each class (OS longer than or equal to 12 months *versus* shorter than 12 months in the case) by maximizing the difference between the class centroids and minimizing the within-class variance when the data being projected onto the DF (Rhys, 2020).

The SVM and the RF algorithms are more alike “black-box” models. The SVM algorithm finds an optimal linear hyperplane that best separates the two classes and is penalized for having cases inside its decision boundary defined by the support vectors. The algorithm can also add a kernel, namely, an extra dimension, to deform the feature space, so that a linear hyperplane can separate the classes (Rhys, 2020). The RF algorithm is an implementation of a bagging technique for decision tree algorithm. It randomly samples cases and features to create a large number of tree classifiers on a binary prediction task that are highly uncorrelated. Then, new data are passed to the trees to make their own prediction, and the model prediction is made based on the majority of the predictions from each tree (Rhys, 2020).

Global surrogate is a common global model-agnostic method to interpret a black box model (e.g., SVM or RF model) by using a surrogate model with a good intuition. In this case, we train a linear regression model or a decision tree model to fit the black box-predicted probability or response, respectively. R-square was used to measure how close the surrogate model is to the black box model (Elshawi et al., 2019).

## Results

As shown in Figure 1, 399 GC patients developing metastasis or recurrence after curative intent gastrectomy were enrolled for modeling. The median survival after metastasis was 8.2 months. Two lost to follow-up cases were removed. Fourteen patients were still alive (survival time ranged from 20.7 to 144.0 months, median 95.1 months), so all the living patients had an OS of no less than 12 months and were assigned to the negative subgroup. Of 385 patients who reached the endpoint (mOS = 7.8 months), 143 patients survived for no less than 12 months (negative subgroup). Overall, the negative subgroup consisted of 157 cases, and the positive subgroup (post-metastatic survival time <12 months) consisted of 242 cases. The ratio of the majority to the minority was 1.54:1. The five most frequent metastasis sites were 46.1% for distant lymph nodes (*n* = 184), 26.8% for the liver (n = 107), 19.8% for the peritoneum (n = 79), 15.8% for bone (*n* = 63), and 15.5% for the chest (n = 62). The baseline information is briefly presented in Table 1. No missing value existed.

**TABLE 1:** Baseline characteristics of the patients enrolled for modeling.

	Overall (n = 399)	Negative (n = 157)	Positive (n = 242)	*p*-value
General information				
Sex (male)	283 (70.93)	108 (68.79)	175 (72.31)	0.519
Age at surgery, y	62.00 (54.00, 68.50)	60.00 (51.00, 68.00)	64.00 (56.00, 69.00)	0.012
DFS, mo	11.87 (5.94, 21.72)	11.43 (4.40, 21.70)	12.02 (6.56, 21.70)	0.378
Age at first metastasis, y	64.00 (55.00, 70.00)	62.00 (53.00, 69.00)	65.00 (56.00, 71.00)	0.014
OS, mo	8.23 (3.77, 17.20)	21.40 (14.90, 31.13)	4.46 (2.51, 7.34)	<0.001
Pathological information				
T stage				0.048
T1	12 (3.01)	6 (3.82)	6 (2.48)	
T2	26 (6.52)	17 (10.83)	9 (3.72)	
T3	231 (57.89)	89 (56.69)	142 (58.68)	
T4a	105 (26.32)	38 (24.20)	67 (27.69)	
T4b	21 (5.26)	7 (4.46)	14 (5.79)	
Tx	4 (1.00)	0 (0.00)	4 (1.65)	
N stage				<0.001
N0	59 (14.79)	38 (24.20)	21 (8.68)	
N1	81 (20.30)	41 (26.11)	40 (16.53)	
N2	105 (26.32)	28 (17.83)	77 (31.82)	
N3a	112 (28.07)	36 (22.93)	76 (31.40)	
N3b	39 (9.77)	14 (8.92)	25 (10.33)	
Nx	3 (0.75)	0 (0.00)	3 (1.24)	
Grade				0.093
G1	5 (1.25)	2 (1.27)	3 (1.24)	
G2	84 (21.05)	43 (27.39)	41 (16.94)	
G3	273 (68.42)	98 (62.42)	175 (72.31)	
G4	5 (1.25)	3 (1.91)	2 (0.83)	
Gx	32 (8.02)	11 (7.01)	21 (8.68)	
Location^¶^				
Cardia	223 (55.89)	93 (59.24)	130 (53.72)	0.327
Body	137 (34.34)	51 (32.48)	86 (35.54)	0.603
Pylorus	125 (31.33)	44 (28.03)	81 (33.47)	0.301
Linitis plastica	4 (1.00)	1 (0.64)	3 (1.24)	>0.999
Histology^¶^				
Adenocarcinoma, NOS	333 (83.46)	136 (86.62)	197 (81.40)	0.218
Mucinous adenocarcinoma	54 (13.53)	14 (8.92)	40 (16.53)	0.043
SRC	25 (6.27)	7 (4.46)	18 (7.44)	0.323
Borrmann type				0.819
I	18 (4.51)	8 (5.10)	10 (4.13)	
II	111 (27.82)	42 (26.75)	69 (28.51)	
III	223 (55.89)	93 (59.24)	130 (53.72)	
IV	25 (6.27)	9 (5.73)	16 (6.61)	
Unknown	22 (5.51)	5 (3.18)	17 (7.02)	
Treatment information				
Resection site				0.731
Proximal	16 (4.01)	8 (5.10)	8 (3.31)	
Distal	101 (25.31)	35 (22.29)	66 (27.27)	
Total	273 (68.42)	112 (71.34)	161 (66.53)	
Others	9 (2.26)	2 (1.27)	7 (2.89)	
Procedure				0.557
Open gastrectomy	364 (91.23)	141 (89.81)	223 (92.15)	
Laparoscopic gastrectomy	34 (8.52)	16 (10.19)	18 (7.44)	
Unknown	1 (0.25)	0 (0.00)	1 (0.41)	
Lymphadenectomy				0.695
D1	128 (32.08)	54 (34.39)	74 (30.58)	
D2	212 (53.13)	83 (52.87)	129 (53.31)	
Unknown	59 (14.79)	20 (12.74)	39 (16.12)	
Radiotherapy (yes)	4 (1.00)	1 (0.64)	3 (1.24)	0.939
Adjuvant chemotherapy (yes)	298 (74.69)	116 (73.89)	182 (75.21)	0.858
Adjuvant chemotherapy cycles^§^	5 (3, 6)	5 (3, 6)	5 (3, 6)	0.290
Palliative chemotherapy (yes)	317 (79.45)	137 (87.26)	180 (74.38)	0.003
First-line drugs^¶^				
Platinum	45 (11.28)	26 (16.56)	19 (7.85)	0.012
Fluorouracil	72 (18.05)	33 (21.02)	39 (16.12)	0.267
Taxane	28 (7.02)	14 (8.92)	14 (5.79)	0.319

Abbreviations: DFS, disease-free survival; SRC, signet-ring cell.

^¶^ In this variable, the count of each category did not sum up to the total number of cases within a subgroup due to overlapping distribution; therefore, a chi-squared or Fisher’s exact test was conducted within each row. Otherwise, the test for categorical variables was conducted within each matrix.

^§^ Only the patients who had a history of adjuvant chemotherapy were summarized.

Continuous variables were presented by the median (interquartile range).

After excluding some features with massive missingness, a total of 62 laboratory indexes were in the feature space, and the distribution of missing values was generally at random (Supplementary Figure S1A). No clear pattern was observed when comparing these indexes between the positive and the negative subgroups (Supplementary Figure S1B,C). The feature filter process returned a feature list ordered by the RF importance and then passed it into further modeling. Interestingly, the highly important features were generally the laboratory information at metastasis, i.e., inflammatory index, blood cell test, and biochemistry. Supplementary Figure S1C illustrates the 30 most important features for brevity.

After tuning the optimal number of the features and the hyperparameters, the model performances are shown in Table 2. The LDA model had the highest AUROC (m ± sd; 0.78 ± 0.021), followed by the SVM model with an AUROC of 0.77 ± 0.014 and the RF model with an AUROC of 0.77 ± 0.0064. The SVM model ranked first with respect to recall (0.93 ± 0.031), and the RF model ranked first with respect to precision (0.80 ± 0.026). So, we further look into these three models.

**TABLE 2:** Comparison of model performance across different machine learning algorithms for predicting 12-month survival in patients with metastatic gastric cancer.

Algorithm	AUROC	Recall	Precision	F1-score	Accuracy
LR	0.68 ± 0.055	0.76 ± 0.058	0.69 ± 0.083	0.72 ± 0.033	0.68 ± 0.045
ER	0.75 ± 0.006	0.74 ± 0.031	0.76 ± 0.037	0.75 ± 0.033	0.70 ± 0.041
KNN	0.75 ± 0.014	0.85 ± 0.039	0.74 ± 0.014	0.79 ± 0.017	0.73 ± 0.018
LASSO	0.76 ± 0.016	0.74 ± 0.027	0.76 ± 0.046	0.75 ± 0.035	0.70 ± 0.046
LDA	**0.78 ± 0.021**	0.84 ± 0.018	0.78 ± 0.023	0.81 ± 0.016	0.76 ± 0.023
RF	0.77 ± 0.006	0.82 ± 0.039	**0.80 ± 0.026**	0.81 ± 0.019	0.76 ± 0.022
RR	0.76 ± 0.016	0.74 ± 0.027	0.76 ± 0.046	0.75 ± 0.035	0.70 ± 0.046
SVM	0.77 ± 0.015	**0.93 ± 0.031**	0.72 ± 0.02	0.81 ± 0.015	0.74 ± 0.022
XGBoost	0.73 ± 0.035	0.84 ± 0.162	0.72 ± 0.037	0.77 ± 0.092	0.70 ± 0.083

Abbreviations: AUROC curve, area under the receiver operating characteristic curve; LR, logistic regression; ER, elastic-net regression; KNN, K-nearest neighbor; LASSO, least absolute shrinkage and selection operator; LDA, linear discriminant analysis; RF, random forest; RR, ridge regression; SVM, support vector machine.

The bold value indicates the highest performance in the column.

**FIGURE 2:**
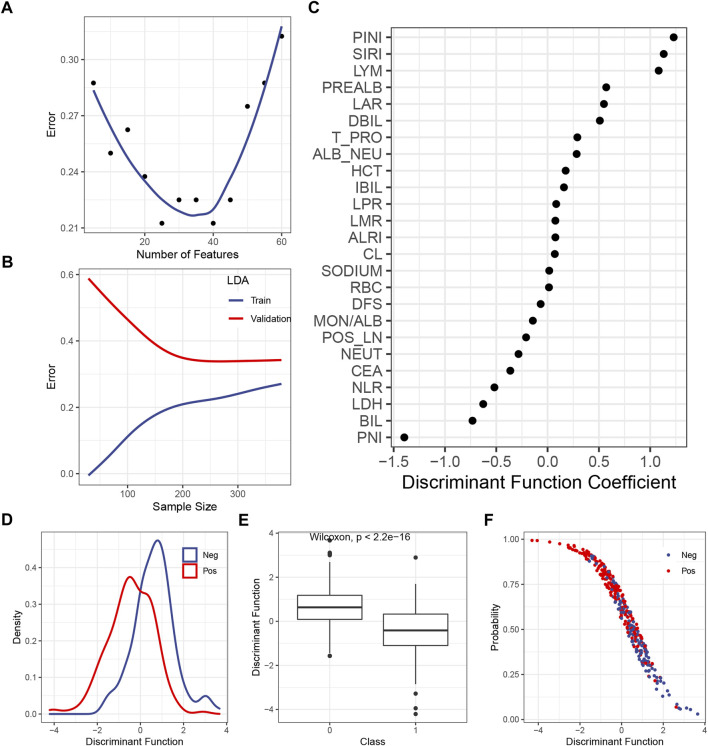
Construction of the LDA model. **(A)** Correlation between the predicted error in the validation set and the number of features. **(B)** Learning curve of the LDA model. **(C)** DF coefficient of the features in the model. All the features were scaled, so the absolute value of the coefficient reflected the contribution of a feature to the model. **(D)** Distribution of the DF in the positive or the negative subgroup. **(E)** Difference of the median DF in each subgroup was examined by the Wilcoxon test. **(F)** Plot of the DF over the predicted probability of being positive by the final LDA model. Abbreviations: LDA, linear discriminant analysis; DF, discriminant function; PINI, prognostic inflammatory nutrition index; SIRI, systemic inflammation response index; LYM, lymphocyte; PREALB, prealbumin; LAR, lymphocyte–albumin ratio; DIBL, direct bilirubin; T_PRO, total protein; NEU, neutrophil; HCT, hematocrit; IBIL, indirect bilirubin; LPR, lymphocyte platelet ratio; LMR, lymphocyte–monocyte ratio; ALRI, aspartate aminotransferase–lymphocyte ratio index; RBC, red blood cell; DFS, disease-free survival; MON, monocyte; ALB, albumin; POS_LN, number of positive lymph node; CEA, carcinoembryonic antigen; NLR, neutrophil–lymphocyte ratio; LDH, lactic dehydrogenase; BIL, bilirubin; PNI; prognostic nutrition index.

The LDA itself had no hyperparameter to be tuned, so only the number of the included features should be tested. Figure 2A shows that the inclusion of the first 25 or 40 features yielded the lowest error in the validation set, so 25 was chosen to make the model simpler. The learning curve illustrated that, with 25 features being considered, the gap between the train and the validation error became steady as the sample size exceeded 200 (Figure 2B). The DF coefficient for each included feature is shown in Figure 2C. The absolute value of the coefficient reflected the contribution of the feature to the model. By summing up the product of the DF coefficient and the feature, a DF was calculated for each case. The distribution of the DF in each class was a bell-shaped curve, with a clearly distinct summit from each other (Figure 2D). As expected, the mean of the DF in each class was statistically different (Figure 2E). The plot of the DF (*x*-axis) over the probability of being predicted as positive by the final model (*y*-axis) demonstrated that, as the DF became greater, the probability of being predicted as positive declined (Figure 2F), which was consistent with the DF distribution across the classes (Figure 2E).

**FIGURE 3:**
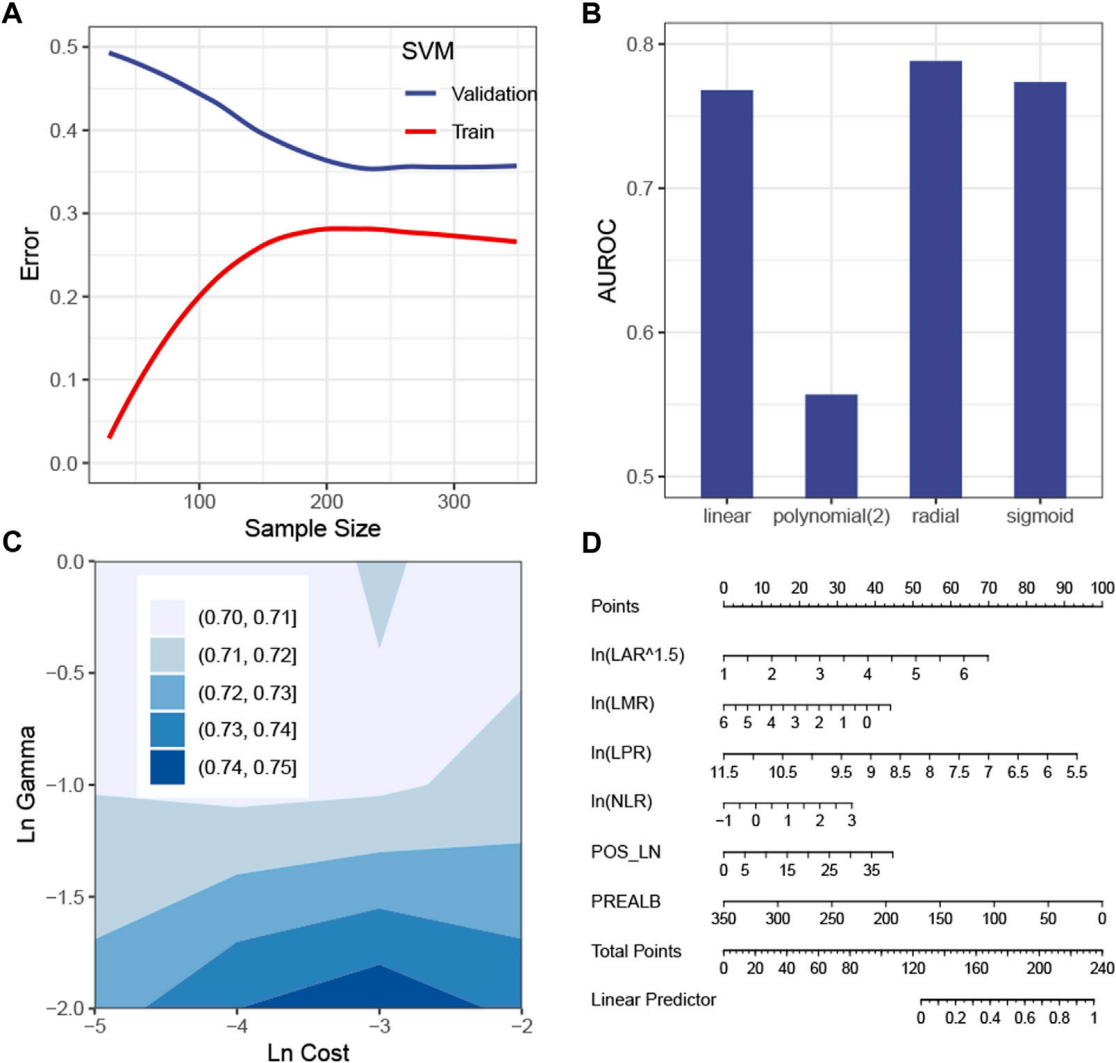
Construction of the SVM model. **(A)** Learning curve of the SVM model. **(B)** Simulation test of the tuning *kernel* function measured by the AUROC. **(C)** Simulation test of tuning *cost* and *gamma* measured by the AUROC. **(D)** Global surrogate model visualized by a nomogram to approximate the predicted probability of being positive by the SVM model. Abbreviations: SVM, support vector machine; AUROC curve, area under the receiver operating characteristic curve; LAR, lymphocyte–albumin ratio; LMR, lymphocyte–monocyte ratio; LPR, lymphocyte–platelet ratio; NLR, neutrophil–lymphocyte ratio; POS_LN, number of positive lymph node; PREALB, prealbumin.

The SVM model with the tuned hyperparameters (Supplementary Table S2) performed best with respect to recall. Figure 3A shows that the sample size was sufficient to stabilize the validation error from the SVM model. Figures 3B,C simulate hyperparameter tuning by grid search, showing that a combination of a radial *kernel* function, a natural log-transformed *cost* of about -3 and a natural log-transformed *gamma* of about -2, gave the highest AUROC in the validation set. It was consistent with the configuration of the actual model tuned by random search (Supplementary Table S2). Because this model only picked the first eight features, it is possible and more intuitive to interpret the SVM model by a linear regression model visualized by a nomogram (Figure 3D). Of the eight features, the linear model, namely, the surrogate model, automatically chose six features by a stepwise method (Figure 3D). The R-square was 0.68.

**FIGURE 4:**
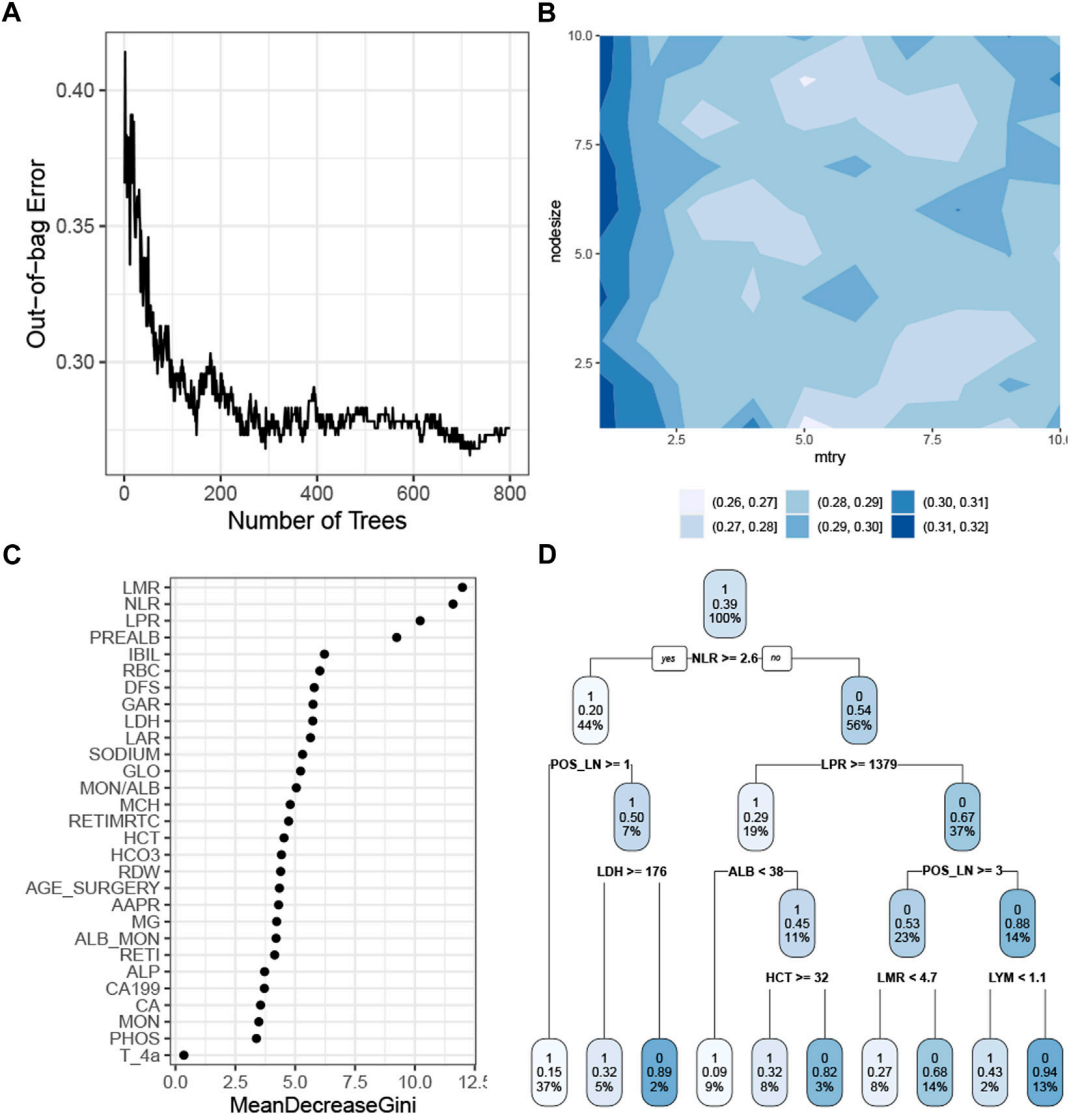
Construction of the RF model. **(A)** Correlation of the out-of-bag error with the number of trees. **(B)** Simulation test of tuning *mtry* and *nodesize* measured by the AUROC. **(C)** Feature importance as measured by the RF algorithm. **(D)** Global surrogate model visualized by a decision tree to approximate the predicted response by the RF model. Abbreviations: RF, random forest; AUROC curve, area under the receiver operating characteristic curve; LMR, lymphocyte–monocyte ratio; NLR, neutrophil–lymphocyte ratio; PREALB, prealbumin; IBIL, indirect bilirubin; RBC, red blood cell; DFS, disease-free survival; GAR, γ-glutamyl transpeptadase–albumin ratio; LDH, lactic dehydrogenase; LAR, lymphocyte–albumin ratio; GLO, globulin; MON, monocyte; ALB, albumin; MCH, mean corpuscular hemoglobin; RETIMRTC, moderate fluorescence–reticulocyte ratio; HCT, hematocrit; RDW, red cell distribution width; AAPR, albumin-to-alkaline phosphatase ratio; RETI, reticulocyte count; ALP, alkaline phosphatase; PHOS, phosphorus.

The RF model with the tuned hyperparameters (Supplementary Table S2) had the highest precision. Figure 4A shows that the model performance stabilized when the number of trees was greater than 400 and reached the best when approximately 700 trees were aggregated. The simulation test showed the optimal combination of the *mtry* and the *nodesize* by grid search (Figure 4B). The rank of the feature demonstrated that the baseline white cell count, platelet count, and prealbumin were the most crucial predictors in the RF model (Figure 4C). This model chose 29 features, so it was more appropriate to use a decision tree algorithm, with a *cp* of 0 and a *maxdepth* of 4, as a surrogate model to approximate the response predicted by the RF model (Figure 4D). The R-square was 0.57.

## Discussion

This study demonstrates that the LDA, SVM, and RF models outperform other algorithms in predicting the survival of patients with metastatic GC. The model performance assessed by a single holdout CV is not reliable because of the effect of randomness in splitting data into training and validation sets. Here, this issue is addressed by repeating the holdout CV five times, so the performance is unbiased in the local cohort, as measured by AUROC, recall, and precision. All the AUROCs of the three models exceed 0.75, which is higher than those in the previous reports (Koo et al., 2011; Custodio et al., 2017; Ma et al., 2021). In addition, our earlier work selected the patients with AGC receiving first-line chemotherapy from the same local cohort and compared the performances of seven survival prediction models on it, showing the best model having an AUROC of 0.60 (Xu et al., 2021). The present study does not impose restrictions on treatment or other factors, so the population is of wider heterogeneity, which may help the model generalize well due to closely reflecting the characteristics of the patients in a real-world setting.

The LDA model performs the best as measured by the AUROC. This model is relatively the most interpretable model among the algorithms tested because it constructs a single and intuitive DF from the original features for classification, which is similar to the well-known logistic regression. In contrast, the SVM and RF models are less explainable; however, they perform the best as measured by recall and precision, respectively. In clinical practice, if we want to identify as many patients as possible with less than 1-year survival by the model, i.e., avoid a false-negative event, we should consider the SVM model because it has the highest recall. If we want to be confident with the ML’s prediction, i.e., avoid a false-positive event, we should consider the RF model due to its highest precision. Therefore, model selection is closely related to the specific practical scenario.

Almost all the features included for modeling are the baseline index of systemic inflammation and malnutrition, albeit with a few exceptions, which is in line with the previous studies. For patients receiving first-line treatment, Kim et al. (2020) identified the clinically relevant features as NLR, neutrophil count, alkaline phosphatase, albumin, lymphocyte count, and white blood cell count. The AGAMENON nomogram weights the neutrophil-to-lymphocyte ratio (NLR) greater than tumor differentiation, metastasis site, or HER2+-treated (Custodio et al., 2017). Hsieh et al. (2016) selected NLR, modified Glasgow prognostic score (mGPS), and Patient-Generated Subjective Global Assessment (PG-SGA) as the most relevant predictors (all of them are inflammation- or nutrition-based scores), compared with age, physical status, differentiation, and metastasis site. In fact, the laboratory index is frequently adopted in models for predicting the survival of metastatic GC patients (Chau et al., 2004; Lee et al., 2007; Kim et al., 2008; Takahari et al., 2014; Wang et al., 2016; Kim et al., 2020), as extra information added on the well-recognized predictors. The present study further emphasizes the prediction value of the index in a setting of a more heterogeneous population with GC. In other words, the ML model “considers” that the post-metastasis survival is mainly attributed to the characteristics of the patients at the time of metastasis, other than the history of staging, surgery, adjuvant chemotherapy, and so on. This may explain the prognoses of the patients with metachronous or synchronous GC being the same as long as the cancer is at an advanced stage (Patel et al., 2007).

The current study has several limitations. First, the models are confident to generalize well in the local population because they are unbiasedly evaluated by repeated CV; however, the case is uncertain in other situations due to lack of external validation. Second, the ensemble technique, a common method to enhance model performance (Rhys, 2020), is not utilized for modeling. We consider there is a trade-off between model complexity and model performance: using ensemble is very likely to improve the model at the cost of long running time and poor interpretability and vice versa. So, here we prefer an easier model, at the cost of performance, in order to facilitate real clinical practice. Third, there are still over 20 features in some models, albeit feature filtering has been conducted. This would impede the models from being used as a quick screening tool for practitioners.

In conclusion, on the basis of the readily available information from the EMR and LIS, the mainstream ML method can produce satisfactory models for predicting survival in patients with metastatic GC who experienced prior radical gastrectomy. The algorithm should be selected according to the measurement and its meaning in a practical scenario.

## Data Availability

The raw data supporting the conclusion of this article will be made available by the authors, without undue reservation.
