# Supplementary material for: Machine learning models for predicting one-year survival in patients with metastatic gastric cancer who experienced upfront radical gastrectomy
Source: Front Mol Biosci. 2022 Dec 1;9:937242. doi: 10.3389/fmolb.2022.937242 (PMC9751187; doi:10.3389/fmolb.2022.937242)
Supplement: Supplementary file 1 [file DataSheet1.docx]

Supplementary Material

# Supplementary Data

# Supplementary Figures and Tables

## Supplementary Tables

| **Supplementary Table 1. The entire feature space for modelling** |
| --- |
| **General information** |
| Age of surgery (yrs), age of metastasis (yrs), progression free survival (mo), sex, palliative chemotherapy (yes vs. no), postoperative adjuvant chemotherapy (yes vs. no), number of the cycles of the adjuvant chemotherapy, basic disease (yes vs. no), hypertension (yes vs. no), diabetes (yes vs. no), cardiovascular diseases (yes vs. no), chronic liver diseases (yes vs. no), chronic respiratory diseases (yes vs. no), surgery history (yes vs. no), site of metastasis, number of metastasis, survival status, survival time. |
| **Pathological information** |
| Primary tumor location, histology, tumor size, grade, Borrmann type, T stage, involved organ when T4b was met, N stage, number of the examined lymph nodes, number of the positive lymph nodes, AJCC 8^th^ stage, vessel invasion (yes vs. no), lymphatic invasion (yes vs. no), nerve invasion (yes vs. no), tumor nodule (yes vs. no), HER2 positive (yes vs. no). |
| **Surgical information** |
| Resection site, surgery procedure, resection of other organs, extent of lymphadenectomy, anastomosis. |
| **First-line chemotherapy information** |
| Administration of platinum (yes vs. no), fluorouracil (yes vs. no), taxane (yes vs. no), irinotecan (yes vs. no), targeted drugs (yes vs. no); regimen of platinum and fluorouracil (yes vs. no), regimen of platinum, fluorouracil and taxane (yes vs. no), regimen of fluorouracil and taxane (yes vs. no), regimen of platinum and taxane (yes vs. no). |
| **Laboratory records at metastasis** |
| White blood cell count, neutrophil count, lymphocyte count, monocyte count, eosinophil count, basophil count, red blood cell count, hematoglobin, red blood cell specific volume, mean corpuscular volume, mean corpuscular hemoglobin, mean corpuscular hemoglobin concentration, red cell distribution width, red cell distribution width standard deviation, platelet, platelet distribution width, platelet percent, mean platelet volume, reticulocyte count, immature reticulocyte fraction, low fluorescence reticulocyte ratio, moderate fluorescence reticulocyte ratio, high fluorescence reticulocyte ratio, total protein, albumin, globulin, total bilirubin, direct bilirubin, indirect bilirubin, bile acid, alanine transaminase, aspartate aminotransferase, γ-glutamyl transpeptadase, alkaline phosphatase, lactic dehydrogenase, prealbumin, creatinine, urea nitrogen, uric acid, potassium, sodium, chloride, bicarbonate, calcium, phosphorus, manganese, glucose, carbohydrate antigen 19-9, carcinoembryonic antigen, platelet lymphocyte ratio (PLR), neutrophil lymphocyte ratio (NLR), lymphocyte monocyte ratio (LMR), systemic inflammation response index (SIRI), systemic immune-inflammation index (SII), prognostic nutritional index (PNI), platelet albumin ratio (PAR), prognostic inflammatory nutrition index (PINI), albumin - monocyte, monocyte albumin ratio, albumin - neutrophil, albumin to alkaline phosphatase ratio (AAPR), lymphocyte albumin ratio (LAR), aspartate aminotransferase lymphocyte ratio index (ALRI), γ-glutamyl transpeptadase albumin ratio (GAR) |

| **Supplementary Table 2. Hyperparameter tuning list for each algorithm** | | |
| --- | --- | --- |
| Algorithm | Tuned hyperparameter  (by random search) | Model performance  (AUROC) |
| KKNN | N_feat = 22 | 0.761 |
|  | Kernal = “epanechnikov” |  |
|  | K = 43 |  |
| LDA | N_feat = 25 | 0.777 |
| RR | N_feat = 36 | 0.761 |
|  | Lambda = 2.27e-28 |  |
| LASSO | N_feat = 26 | 0.761 |
|  | Lambda = 2.272563e-28 |  |
| ER | N_feat = 23 | 0.753 |
|  | Lambda = 1.452625e-28 |  |
|  | Alpha = 0.3735086 |  |
| SVM | N_feat = 8 | 0.772 |
|  | Kernal = “radial” |  |
|  | Cost = 0.04639265 |  |
|  | Gamma = 0.2829212 |  |
| RF | N_feat = 29 | 0.769 |
|  | N_tree = 713 |  |
|  | Min_node_size = 9 |  |
|  | Mtry = 5 |  |
| XGBOOST | N_feat = 26 | 0.728 |
|  | Eta = 0.03821941 |  |
|  | Gamma = 2.3673324 |  |
|  | Max_depth = 3 |  |
|  | Min_child_weight = 1.178486451 |  |
|  | Colsample_bytree = 0.9056158 |  |
|  | Subsample = 0.6787385 |  |
|  | Nrounds = 14 |  |
| Abbreviations: AUROC, area of receiver operating characteristics curve; N_feat, number of features; KKNN, kernal k-nearst neighbour; LDA, linear discriminant analysis; RR, ridge regression; LASSO, least absolute shrinkage and selection operator; ER, elastic regression; SVM, support vector machine; RF, random forest. | | |

## Supplementary Figures


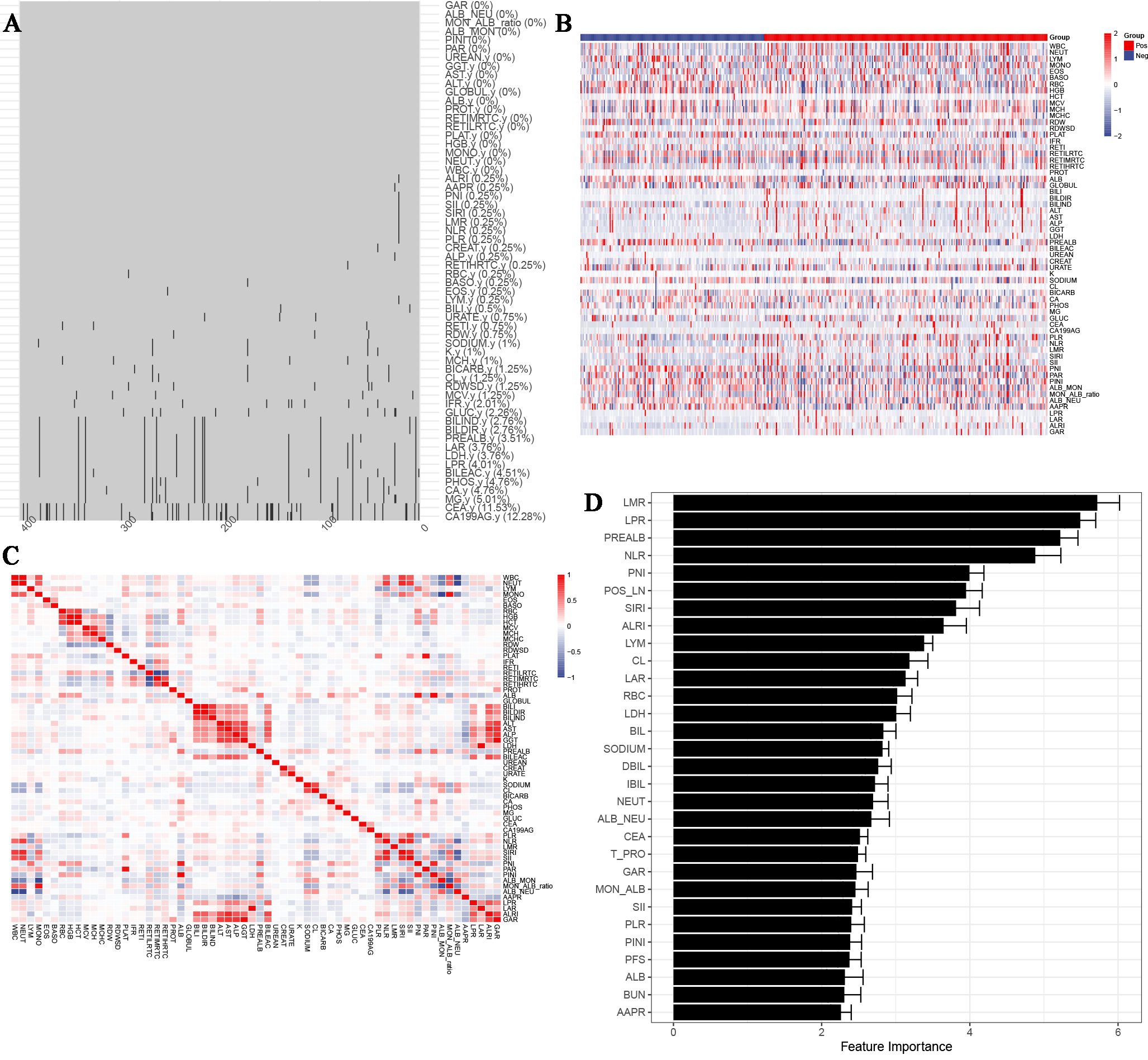


**Supplementary Figure 1.** Feature proprocessing and visualization. (A) The profile of missing value in the laboratory information. (B) The relative level of each laboratory index across the entire cohort. Each index was scaled. (C) The correlation of each index. (D) The feature rank as measured by the random forest algorithm in the feature filter process. Only the 30 most top-ranked features were displayed for brevity.
